# Horizontal transfer and evolution of wall teichoic acid gene cassettes in *Bacillus subtilis*

**DOI:** 10.12688/f1000research.51874.1

**Published:** 2021-05-07

**Authors:** Granger Sutton, Gary B. Fogel, Bradley Abramson, Lauren Brinkac, Todd Michael, Enoch S. Liu, Sterling Thomas

**Affiliations:** 1 J. Craig Venter Institute, Rockville, Maryland, 20850, USA; 2 Natural Selection, Inc., San Diego, CA, 92121, USA; 3 The Salk Institute for Biological Studies, La Jolla, CA, 92037, USA; 4 Noblis, Inc., Reston, VA, 20191, USA

**Keywords:** wall teichoic acids, pan-genome, pan-genome graph, core genes, Bacillus subtilis

## Abstract

**Background:** Wall teichoic acid (WTA) genes are essential for production of cell walls in gram-positive bacteria and necessary for survival and variability in the cassette has led to recent antibiotic resistance acquisition in pathogenic bacteria.

**Methods:** Using a pan-genome approach, we examined the evolutionary history of WTA genes in *Bacillus subtilis* ssp. *subtilis*.

**Results:** Our analysis reveals an interesting pattern of evolution from the type-strain WTA gene cassette possibly resulting from horizontal acquisition from organisms with similar gene sequences. The WTA cassettes have a high level of variation which may be due to one or more independent horizontal transfer events during the evolution of *Bacillus subtilis* ssp. *subtilis*. This swapping of entire WTA cassettes and smaller regions within the WTA cassettes is an unusual feature in the evolution of the *Bacillus subtilis* genome and highlights the importance of horizontal transfer of gene cassettes through homologous recombination within *B. subtilis* or other bacterial species.

**Conclusions:** Reduced sequence conservation of these WTA cassettes may indicate a modified function like the previously documented WTA ribitol/glycerol variation. An improved understanding of high-frequency recombination of gene cassettes has ramifications for synthetic biology and the use of *B. subtilis* in industry.

## Introduction

In a recent paper, we studied the relationship between essential and core genes in *Bacillus subtilis* ssp. *subtilis* through a pan-genomic approach. Core genes are the set of genes present in all or almost all strains of a species/subspecies in a pan-genome. Pan-genomes are determined computationally by finding orthologous gene clusters (OGC) between strains based on homology and genome context. ^1– 3^ An OGC is the set of genes, with at most one per strain, that have been computationally determined to be orthologs. An OGC is “core” if the number of genes in the OGC exceeds or equals some threshold such as 95% or 100% of the strains in the pan-genome. A node within a pan-genome graph (PGG) is an OGC and if two nodes (OGCs - specifically the genes in the OGCs) are adjacent in the genome of one or more of the pan-genome strains this is considered an edge. While core genes are determined computationally, “essential genes” are experimentally determined genes that render an organism as nonviable if removed in laboratory growth conditions. Such genes are determined experimentally through knockout studies via methods such as random transposon insertion.

For example, Koo *et al*. ^4^ and Kobayashi *et al*. ^5^ computationally and experimentally determined the essential gene set in the gram-positive bacterium *Bacillus subtilis* ssp. *subtilis.* For *B. subtilis* ssp. *subtilis*, both Koo *et al*. ^4^ and Kobayashi *et al*. ^5^ used similar single knockout methods to determine “essential” genes when grown in LB at 37°C. Koo identified 257 essential genes while Kobayashi identified 271 essential genes. The union of these two sets resulted in 305 essential genes. ^6^ Sutton *et al*. ^6^ mapped these 305 genes to the PGG OGCs using the type strain 168 RefSeq genome NC_000964.3 (BioSample SAMEA3138188) used by Koo *et al*. ^4^ and Kobayashi *et al*. ^5^ and 289 were determined to be core OGCs. ^6^ For those genes that were determined to be essential in laboratory conditions but determined not to be core for the subspecies pan-genome, Sutton *et al*. ^6^ found that some were not truly essential but rather “conditionally essential” due to the presence of other genes such as toxin/antitoxin cognate pairs where the antitoxin is essential in the presence of the toxin gene.

Curiously, eight of the 305 essential genes that were not core genes are involved in the biosynthesis of wall teichoic acid (WTA). These include *tuaB* (OGC 4729 present in 85 of 108 genomes), *mnaA/yvyH* (OGC 4735 present in 84 of 108 genomes), *tagH* (OGC 4744 present in 84 of 108 genomes), *tagG* (OGC 4745 present in 35 of 108 genomes), *tagF* (OGC 4746 present in 35 of 108 genomes), *tagD* (OGC 4748 present in 35 of 108 genomes), *tagA* (OGC 4749 present in 35 of 108 genomes) and *tagB* (OGC 4750 present in 35 of 108 genomes). By examining the PGG we determined that genes homologous but diverged from the type strain 168 WTA genes were present at the same relative genome location but were contained in other OGCs. In order to understand the evolution of these essential WTA genes that are not contained in single OGCs, more diverged protein orthology was used to overcome the strong OGC constraint of a minimum of 90% identity over 90% of gene length at the nucleotide level. Our analysis indicates that the WTA genes have a high level of variation possibly due to horizontal gene transfer via recombination as entire cassettes and smaller regions in *B. subtilis* ssp. *subtilis.*

WTA genes are involved in production of anionic glycopolymers required for consistent cell shape and division. ^7, 8^ Mutants deficient in WTA biosynthesis show increased sensitivity to temperature and certain buffer components and cells tend to aggregate in culture. ^9– 14^ While the WTA genes have been shown to be dispensable, ^11^ the resulting cells have abnormal morphology and show impaired growth and reproduction. *B. subtilis* and *S. aureus* mutants deficient in lipoteichoic acid (LTA) biosynthesis can be obtained but only if grown under a narrow range of conditions; they are temperature sensitive and exhibit severe growth defects. ^9, 10^

Taken together, LTAs and WTAs create what has been aptly described by Neuhaus and Baddiley as a “continuum of negative charge” that extends from the bacterial cell membrane beyond the outermost layers of peptidoglycan. ^15^ New pathogenesis-related functions for WTAs have also been realized and it has been suggested that the biosynthetic enzymes that make these polymers are targets for novel antibacterial agents. ^16, 17^ Indeed, the first WTA-active antibiotic ^18^ acts by blocking the export of WTA to the extracellular surface. The chemical structures of WTAs vary in gram-positive bacteria but the most common structures are glycerol or ribitol phosphate repeats. ^19– 26^ *B. subtilis* can make poly (glycerol phosphate) or poly (ribitol phosphate) WTAs depending on the strain, ^27^ while *S. aureus* strains primarily make poly (ribitol phosphate) WTAs. ^28– 31^

The ribitol WTA genes ( *tar*) were thought to distinguish *B. subtilis* ssp. *spizizenii* strains from the glycerol WTA genes ( *tag*) contained in *B. subtilis* ssp. *subtilis* strains based on functional studies in *B. subtilis* strain W23 which is a *B. subtilis* ssp. *spizizenii* strain and *B. subtilis* strain 168 which is a *B. subtilis* ssp. *subtilis* strain. ^32^ Sequencing of the W23 *tar* genes revealed eight open reading frames in two adjacent divergently transcribed operons, *tarABIJKL* and *tarDF*, where *tarA*, *tarB*, *tarD* and *tarF* have clear homology to their counterparts *tagA*, *tagB*, *tagD* and *tagF* while *tarI*, *tarJ*, *tarK* and *tarL* have no obvious homology to *tag* genes. The four conserved *tar/tag* genes appear to construct the basic core of the teichoic acid structure although for *tar/tagF* the function is somewhat different since *tagF* is much longer than and shares only C-terminal homology with *tarF*. ^33^ The four *tar* genes which are not conserved presumably are specific to the ribitol modifications to teichoic acids. The *tag* genes are also organized in two adjacent divergently transcribed operons *tagABC* and *tagDEF* where *tagC* and *tagE* have no obvious homology to *tar* genes. ^32^ More recently it was determined that the ribitol/glycerol distinction of *tar* versus *tag* genes is not distinguishing between *spizizenii* and *subtilis* subspecies but rather either subspecies can contain one or the other. ^34^

## Methods

For the *B. subtilis* ssp. *subtilis* pan-genome we selected strains with complete genomes in RefSeq. ^35^ We restricted our analysis to complete genomes to ensure that missing genes due to incomplete genome sequencing/assembly did not affect the approach or results. We limited our choice to RefSeq for two reasons: RefSeq performs a series of quality checks to remove dubious genome assemblies, and the initial pan-genome construction depends upon reasonably consistent annotation which RefSeq provides. We extracted the genomes based on organism name: *Bacillus subtilis* (we did not specify subspecies, since for many RefSeq genomes a subspecies is not given). For each pan-genome we then compared the genomes using a fast Average Nucleotide Identity (ANI) estimate generated using MASH. ^36^ We used type strains and ANI to determine which of these genomes were the desired organism. We also used ANI to remove very closely related strains to reduce oversampling bias (for example, for the *B. subtilis* type strain, 168, has at least eight genomes in RefSeq). We used GGRaSP ^37^ to choose a single medoid sequence from any complete linkage ANI cluster with a threshold of 0.01% or 1/10,000 base pair difference. The strain 168 medoid genome is the Entrez reference genome for the *B. subtilis* type strain (GenBank sequence AL009126.3, BioSample SAMEA3138188, Assembly ASM904v1 **/**GCA_000009045.1) which can be used to map the Koo *et al*. ^4^ and Kobayashi *et al*. ^5^ results.

Using this approach, for *B. subtilis* 143 genomes were downloaded from RefSeq. Of these 132 genomes were determined to be *B. subtilis* ssp. *subtilis* based on type strains and ANI. The minimum ANI between any pair of the 132 *B. subtilis* ssp. *subtilis* genomes was 97.28% whereas the maximum ANI of any of the 11 other genomes to the 132 genomes was 95.73% providing good separation between the other subspecies. The 132 genomes were reduced to 109 genomes after removing redundant strains. Finally, we removed strain delta6 (BioSample SAMN05150066) because it is known to have been engineered to remove multiple genes. Thus, we were left with 108 *B. subtilis* genomes (Supplementary Table 1).

For *B. subtilis* ssp. *subtilis* the initial pan-genome was based on the RefSeq annotation of these genomes. The pan-genome was generated using the pan-genome pipeline at the J. Craig Venter Institute (JCVI) at the nucleotide level using default parameters with the exception that a minimum of 90% identity and 90% length for pairwise Blast matches were used to prevent possible clustering of non-orthologous genes. ^38^ This produced ortholog gene clusters (OGC) using gene context ^1^ as well as a Pan-Genome Graph (PGG). ^2^ The PGG has two main components: nodes representing OGCs, and edges representing the sequence between genes and the order and orientation of the genes in the genomes. We updated the code repository for the JCVI pan-genome pipeline with a script: iterate_pgg_graph.pl which calls pgg_annotate.pl for the genomes in the existing PGG in order to ensure consistent annotation of the genomes and iterates until the PGG stabilizes. The script pgg_annotate.pl uses an existing PGG to assign regions of a genome to nodes of the graph. This is done by blasting the medoid gene sequence for the OGC the node represents against the genome and then uses Needleman-Wunsch ^39^ to extend the alignment if needed. If there are conflicting blast matches, then the matches are resolved based on which matches are consistent with the structure of the PGG which encapsulates gene context across the entire pan-genome. Once the nodes of the PGG are mapped to each of the genomes in the pan-genome a new version of the PGG is intrinsic and then explicitly extracted. This process is iterated to stability. This ensures that each genome is consistently annotated so that missing genes are not due to inconsistent annotation.

The medoid nucleotide sequence for each of the 144 OGCs found in any of the WTA cassettes was translated into peptide sequences. These 144 peptide sequences were combined into a multifasta file to create a peptide Blast database (makeblastdb -in WTA_prot.fasta -dbtype prot). A peptide level all versus all Blast search ^40^ of these 144 peptide sequences was performed (blastp -query WTA_prot.fasta -db WTA_prot.fasta -out tmp -task blastp -evalue 0.000001 -outfmt "6 qseqid sseqid pident qstart qend qlen sstart send slen evalue bitscore stitle"). Potential protein ortholog matches were retained if the percent identity was ≥40% and the length of the match was ≥80% of the shorter protein. Matches with smaller bit scores than the first match to a protein from the same clade (i.e. a paralog) were not retained. More limited matches were retained for *tagF* matches in an attempt to determine possible orthologs but they were not treated as likely orthologs. The protein ortholog Blast matches are in Supplementary Table 7 and the probable protein orthologs are in Table 1.

**Table 1.** The protein level orthologous OGCs within the WTA cassettes. Column 1 is the gene name/symbol. Column 2 is the set of OGCs determined to be orthologs at the protein level. Column 3 is the number of the 108 strains in the PGG which contain one of the protein level orthologs. Column 4 is OGC medoid sequence RefSeq annotation for one of the protein level orthologs.

Gene	OGCs	Summed OGC Size	Annotation
*tagV*	3713, 4723	108	medoid_4723 Q433_RS17940 polyisoprenyl-teichoic acid-peptidoglycan teichoic acid transferase TagV
*tuaG*	3723, 4724	108	medoid_4724 OB04_RS18145 glycosyltransferase family 2 protein
*tuaF*	3724, 4725	108	medoid_4725 C7M23_RS06445 Teichuronic acid biosynthesis protein tuaF
*tuaE*	3725, 4726	108	medoid_4726 Bateq7PJ16_RS19495 teichuronic acid biosynthesis protein TuaE
*tuaD*	3726, 4727	108	medoid_4727 BKN48_RS07140 UDP-glucose 6-dehydrogenase TuaD
*tuaC*	3727, 4728	108	medoid_4728 C7M27_RS00270 glycosyltransferase family 4 protein
*tuaB*	3728, 4729	108	medoid_4729 BEST7003_RS17430 MOP flippase family protein
*tuaAc*	3729, 4730	108	medoid_4730 C7M26_RS17205 sugar transferase
*lytC*	3731, 4731, 5419	101	medoid_4731 BEST7003_RS17440 N-acetylmuramoyl-L-alanine amidase LytC
*lytB*	3732, 4732, 5420	98	medoid_4732 BEST7003_RS17445 SpoIID/LytB domain-containing protein
*lytA*	3733, 4733, 5421	101	medoid_4733 BEST7003_RS17450 membrane-bound protein LytA
*tagU*	4734, 8738	85	medoid_4734 EQZ01_RS18735 transcription antiterminator LytR
*mnaA*	3737, 4735, 8737	108	medoid_4735 BSK2_RS18135 UDP-N-acetylglucosamine 2-epimerase (non-hydrolyzing)
*gtaB*	3738, 4736	108	medoid_4736 CD007_RS18080 UTP--glucose-1-phosphate uridylyltransferase GalU
*ggaB*	4738, 5996, 7395, 8734	69	medoid_4738 BEST7003_RS17470 poly (glucosyl N-acetylgalactosamine 1-phosphate) glucosyltransferase
*ggaA*	4739, 5429, 5997, 7091, 7396, 8733	76	medoid_4739 BEST7003_RS17475 glycosyltransferase family 2 protein
*tagH*	3740, 4744, 8735	108	medoid_4744 C7M17_RS18530 teichoic acids export ABC transporter ATP-binding subunit TagH
*tagG*	3741, 4745, 5425, 7157	108	medoid_4745 BEST7003_RS17490 teichoic acids export ABC transporter permease subunit TagG
*tagF*	3745, 4746, 5430, 5998, 7397, 8732	87	medoid_4746 BEST7003_RS17495 teichoic acid poly (glycerol phosphate) polymerase
*tagD*	3746, 4748, 5431, 6915, 7624, 8731	107	medoid_4748 BEST7003_RS17505 glycerol-3-phosphate cytidylyltransferase
*tagA*	3747, 4749, 5432, 6918, 8730	107	medoid_4749 BEST7003_RS17510 N-acetylglucosaminyldiphosphoundecaprenol N-acetyl-beta-D- mannosaminyltransferase
*tagB*	3748, 4750, 5433, 6919, 8729	107	medoid_4750 BEST7003_RS17515 teichoic acid glycerol-phosphate primase
*gerBA*	3751, 4752, 5718	108	medoid_4752 CAH07_RS02865 spore germination protein
*gerBB*	3752, 4753	108	medoid_4753 BEST7003_RS17540 spore germination protein GerBA
*tagT*	3755, 4754, 5438	108	medoid_4754 BEST7003_RS17555 polyisoprenyl-teichoic acid--peptidoglycan teichoic acid transferase TagT
*tarQ*	5418, 6914	45	medoid_5418 ETL58_RS18550 (poly)ribitol-phosphate teichoic acid beta-D-glucosyltransferase
	5423, 7623	51	medoid_5423 CAH07_RS02960 glycosyltransferase

**Table 2.** OGC subpatterns for the WTA cassettes across clades I-VII. The OGC subpatterns show some limited recombination within the WTA cassettes but most recombination seems limited to the entire cassette. Column 1 is the region between core OGCs within the WTA cassette. Column 2 is an OGC subpattern. Columns 3-9 indicate the number of strains within a clade that has the given OGC subpattern for that row. The rows are ordered relative to their order in the WTA cassette from core OGC 3712 to core OGC 3756.

Region	OGC SubPattern	Clade I (n = 43)	Clade II (n = 2)	Clade III (n = 3)	Clade IV (n = 1)	Clade V (n = 23)	Clade VI (n = 35)	Clade VII (n = 1)
3712-3721	(3713-3718,9158,3719-3720)					22		
3712-3721	4723	43	2	3	1	1	35	1
								
3722-3749	(3723-3725,9159,3726-3729)					23		
3722-3749	(3730-3735,9341,3736)					5		
3722-3749	(3730-3735)					1		
3722-3749	(3731-3734)					10		
3722-3749	(3731-3734,9341)					1		
3722-3749	(3736,9341)					5		
3722-3749	(3730-3733,9495)					1		
3722-3749	(3737-3748)					23		
3722-3749	(7961)					1		
3722-3749	(4724-4730)	43	2	3	1		35	1
3722-3749	(4731-4736)							35
3722-3749	(10139,4737-4745)							24
3722-3749	(4744-4745)							3
3722-3749	(10139,4737,5994-5996,7091-7093,4743-4745)							1
3722-3749	(10139,5995-5996,7091-7093,4743-4745)							7
3722-3749	(5418)	43						
3722-3749	(6914)		2					
3722-3749	(5419-5421,4734-4735,5422-5424,4736,4744,5425)	43	2					
3722-3749	(4731,7622,4733-4735,5423,7623,4736,4744,5425)			3				
3722-3749	(8738-8737,4736,8736,7961,8735,5425)				1			
3722-3749	(4734-4736,7156,4744,7157)							1
3722-3749	(4738-4741)	15						
3722-3749	(5426-5430)	7						
3722-3749	(5994-5998)	20						
3722-3749	(7395-7397)	1						
3722-3749	(5431)	43						
3722-3749	(7624)			3				
3722-3749	(5432-5433)	43	2					
3722-3749	(6915-6919)		2					
3722-3749	(5434-5437)	43	2	3				
3722-3749	(8734-8729)				1			
3722-3749	(7625-7631)			3	1			
3722-3749	(4746-4751)						35	
								
3750-3753	(3751-3752)	6	2	3	1	13	2	
3750-3753	(4752-4753)	37				10	32	1
3750-3753	(5718,3752)						1	
								
3754-3756	(3755)			1	1	23		
3754-3756	(4754)	37	1	2			35	1
3754-3756	(5438)	6	1					

The extracted nucleotide WTA cassette sequences were placed in a multifasta file. The sequences were aligned using Mafft ^41^ (mafft –reorder WTA_cassette.fasta > WTA_cassette.mafft). The alignment was trimmed of gappy columns using trimal ^42^ (trimal -in WTA_cassette.mafft -out WTA_cassette.trim -gt 1 -fasta). The trimmed alignment was used as input to RaxML ^43^ run from TOPALi v2.5 ^44^ with default parameters to generate a phylogenetic tree.

Phylogenetic trees were generated from the pan-genome using complete linkage hierarchical clustering of pairwise Jaccard distance and genome ANI distances. The resulting trees were rendered using the Interactive Tree of Life (iTOL). ^45– 47^ A linear illustration of the WTA cassettes was generated using the SimpleSynteny tool. ^48^

## Results

For our 108 strain pan-genome (Supplementary Table 1), the WTA genes in all genomes are colocalized and bounded by two core genes: *yvyE* (OGC 3712) and *ribZB* (OGC 3756). For all but one genome, this region is contiguous and therefore can be considered as a cassette. For strain N1-1 (SAMN10225190), the cassette is split into two nearby contiguous pieces due to an inversion between two inverted copies of an IS1182 transposon, one inside the cassette and one outside the cassette. This could be a real rearrangement or an assembly error. For the purpose of our analysis we treated these two pieces as a single cassette as if the inversion had not taken place.

**Figure 1.**
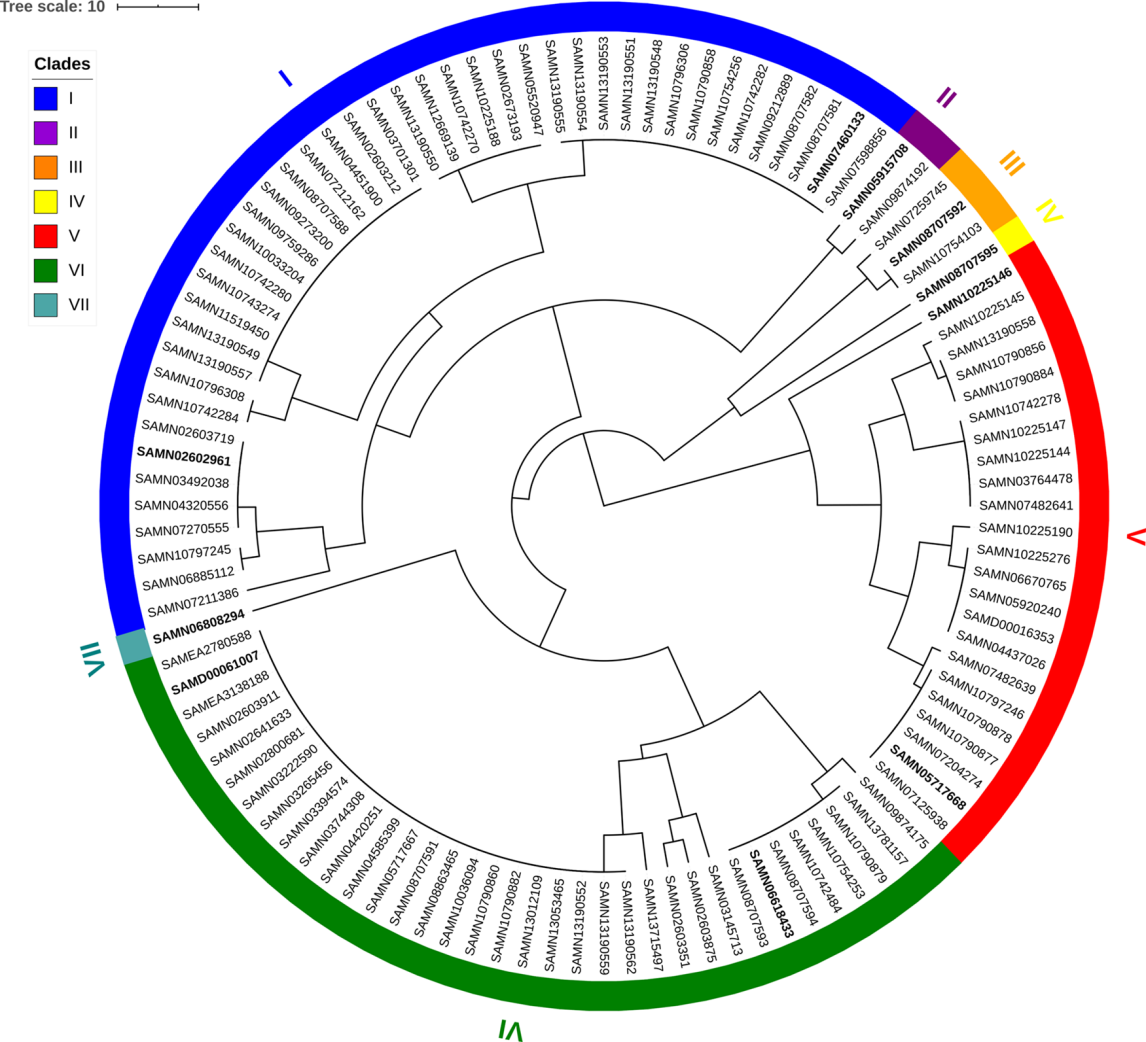
WTA 144 OGC gene content tree based on complete linkage hierarchical clustering of pairwise Jaccard distance of the 144 OGCs in the WTA cassettes. We distinguished seven clades. Clade I (blue) is the ribitol WTA consistent with that found in strain W23. Clade II (purple) and clade III (orange) appear to also be ribitol WTA based on the presence of *tarI* (OGC 5434), *tarJ* (OGC 5435), *tarK* (OGC 5436) and *tarL* (OGC 5437). Clade IV (yellow) may be a ribitol WTA based on a shorter *tagF* gene. Clade V (red) may be a glycerol WTA based on a longer *tagF* gene. Clade VI is the type strain glycerol WTA. Clade VII (teal) is missing many key WTA genes ( *tag/tarBADFG*) so it is unclear how the one strain in that clade is constructing WTAs. The 10 medoid strains used in Figure 3 are in bold.

**Figure 2.**
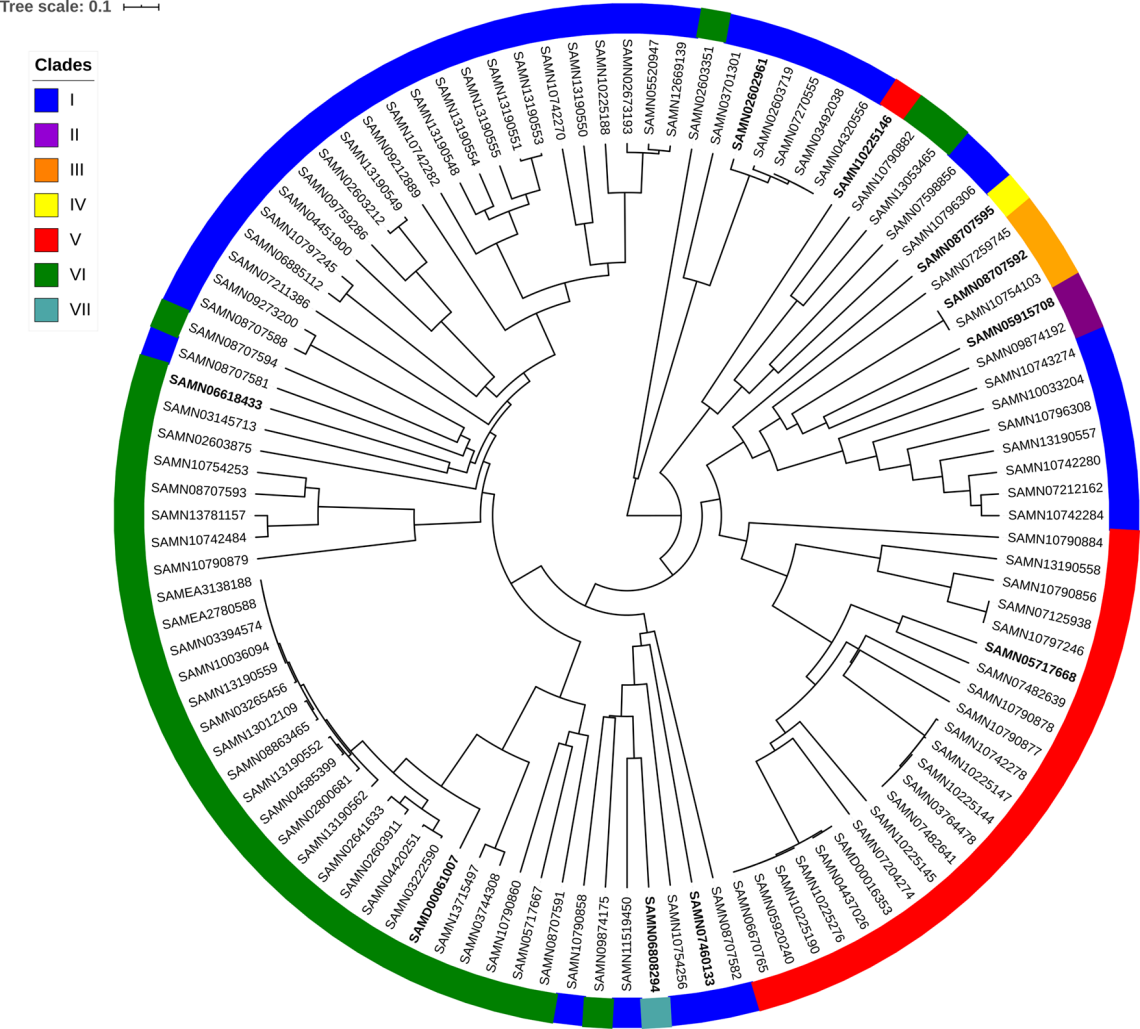
The ANI tree for the 108 strains of *B. subtilis* ssp. ***subtilis*** **in the pan-genome based on complete linkage hierarchical clustering of pairwise genome ANI distances (100 – ANI).** The colors indicate which of the seven WTA gene content clades a strain is in from Figure 1. The 10 medoid strains used in Figure 3 are in bold.

We extracted the PGG annotation, which assigns genome coordinates to OGCs, for just the WTA cassettes from the complete PGG annotation of the 108 genomes (Supplementary Table 2). There were a total of 144 OGCs which were present in one or more of the WTA cassettes which we extracted from the overall set of OGCs (Supplementary Table 3). We also extracted the set of edges between these OGCs from the PGG (Supplementary Table 4). Based on the presence or absence of each of the 144 OGCs in a strain we calculated the pairwise Jaccard distance as a measure of WTA gene content dissimilarity (Supplementary Table 5). The strains were then classified into seven clades (I-VII) based on the Jaccard distance ( Figure 1). Clades I-VII have 43, 2, 3, 1, 23, 35, and 1 strain(s), respectively. To determine whether the gene content difference was due to evolutionary inheritance, we determined the pairwise average nucleotide identity (ANI) similarity between strains (Supplementary Table 6) which is considered to be strongly correlated with evolutionary distance ( Figure 2). Since the WTA gene-content based clades do not form the same clades under ANI distance, but rather intermix, something beyond straight inheritance is accounting for the WTA gene content within strains.

To determine orthology beyond the strict orthology of the OGCs, we used protein level Blast (blastp) to compare the medoid gene sequence of each of the 144 WTA OGCs against the other 143 WTA OGC medoid gene sequences (Supplementary Table 7). The medoid gene sequence of an OGC is the gene sequence with the lowest summed pairwise homology distances as determined by Blast to all other gene sequences in the OGC. The ribitol specific genes *tarI* (OGC 5434), *tarJ* (OGC 5435), *tarK* (OGC 5436) and *tarL* (OGC 5437) had no obvious OGC protein orthologs. Likewise, the glycerol specific genes *tarC* (OGC 4751) and *tarE* (OGC 4747) had no obvious OGC protein orthologs. Based on the ribitol and glycerol specific OGCs, three clades (I, II and III) appear to be ribitol WTAs, one clade (VI) appears to be glycerol WTA, and three clades are an indeterminate WTA type.

**Figure 3.**
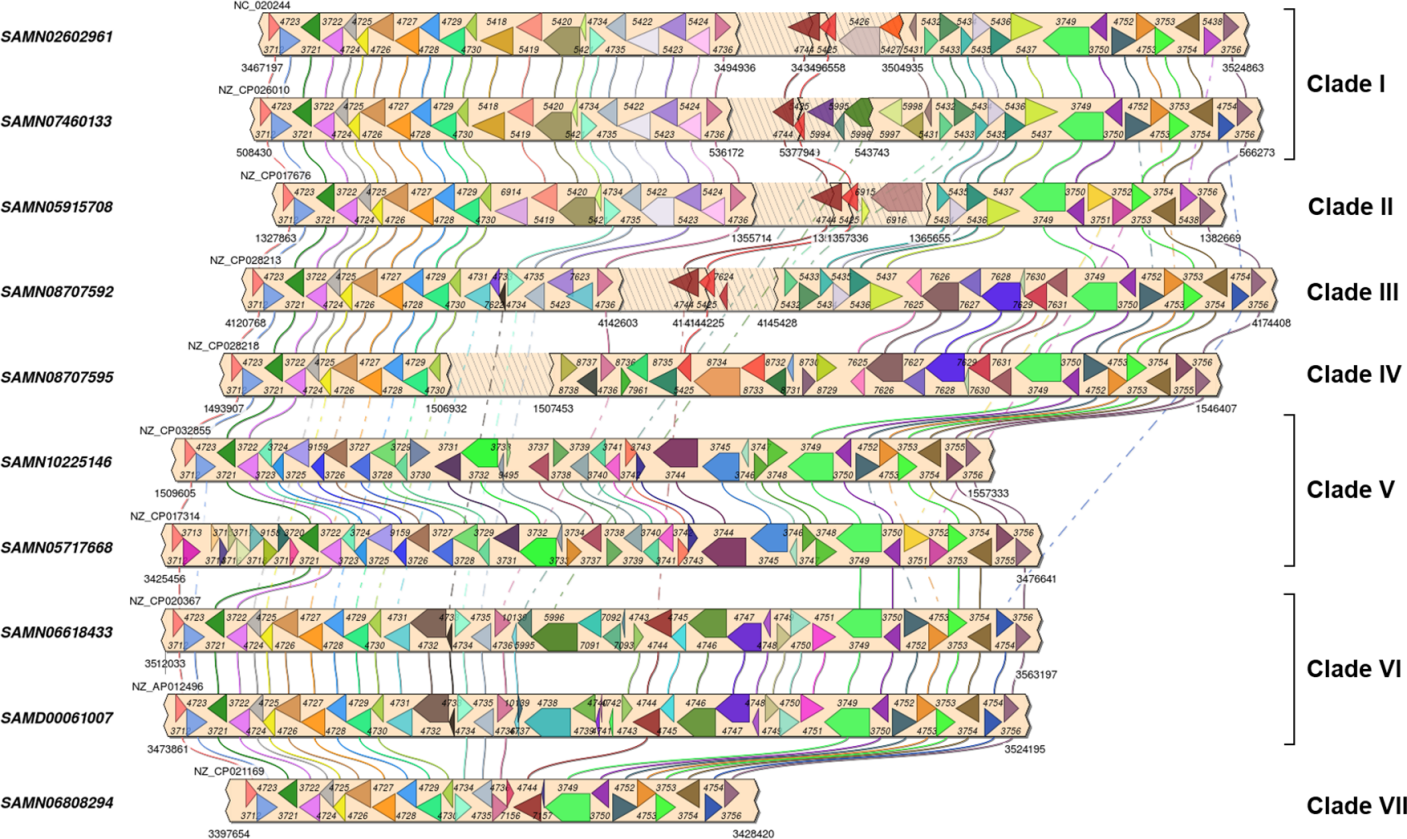
Linear comparison of the WTA cassette of 10 medoid strains representing each of the seven WTA clades (I-VII). Arrows indicate individual WTA genes drawn to scale with order and orientation maintained. The coordinates for the WTA cassettes in SAMN08707592 and SAMN08707595 which are located on the opposite strand, were reversed for rendering. Genes between strains belonging to the same OGC are joined vertically by correponding colored lines.

Within the WTA cassette there are three adjacent OGC pairs (for a total of six OGCs) which are conserved across all 108 strains: *tagO-tuaH* (OGCs 3721-3722), *lytD-pmiA* (OGCs 3749-3750) and *gerBC-ywtG* (OGCs 3753-3754), in that order from bounding OGC 3712 to bounding OGC 3756. Each of these highly similar OGC pairs contain similar DNA sequences which allow for homologous recombination events to occur between strains from different clades. We did not explicitly examine possible recombination between strains of the same clade. We illustrate the basic OGC structure within the WTA cassette for the seven clades shown in Figure 1 in the linear alignment shown in Figure 3 of the WTA cassettes from the 10 medoid genomes determined by ANI and shown in bold in Figure 2.

We examined OGC and OGC protein ortholog based evidence of recombination between clades for each clade. We did this by determining the OGC patterns for each of the 108 strains across the WTA cassette (Supplementary Table 8). An OGC pattern is simply the order of OGCs across the WTA cassette for a given strain from OGC 3712 to OGC 3756. For identical OGC patterns we collapsed identical columns for a simpler presentation in Supplementary Table 9. When analyzing the OGC patterns, we specifically looked for common subpatterns, a subinterval of the OGC pattern, to determine possible recombination events. An OGC pattern or subpattern is shown as a parentheses bounded comma separated list of OGCs where OGCs with consecutive numbers are indicated with a hyphen (e.g. 4724-4730,5418-5421,4734-4735,5422-5424,4736,4744, 5425). A null subpattern indicated by () means there are no OGCs for that subpattern. We looked specifically at the four subregions of the WTA between the bounding OGCs and the three conserved OGC pairs. We designated these four regions based on their bounding OGCs: 3712-3721, 3722-3749, 3750-3753, and 3754-3756.

For the 23 strains in clade V, there are eight OGC patterns (Supplementary Table 9, Supplementary Table 8, Table 2). For 22 strains forming seven OGC patterns all non-core OGCs are specific to clade V with only minor OGC variation in all regions except 3750-3753. For these 22 strains no recombination appears to occur within regions 3712-3721, 3722-3749, and 3754-3756. Within the 3750-3753 region recombination does appear to be occurring with four OGC patterns and 13 strains with OGC subpattern (3751-3752); whereas four OGC patterns and 10 strains have OGC subpattern (4752-4753). In region 3750-3753, there are only three OGC subpatterns for all 108 strains: (3751-3752), (4752-4753), and (5718,3752). Pattern (3751-3752) is in 13/23 clade V strains and 12/85 of other clade strains; whereas, pattern (4742-4753) is in 10/23 clade V strains and 72/85 of other clade strains. This might indicate that OGC subpattern (3751-3752) is ancestral (before recombination) for clade V and OGC subpattern (4752-4753) is ancestral for the other clades. In the 3754-3756 region, there is only one OGC subpattern (3755). Clade V strain PJ-7 (SAMN10225146) is the only clade V strain with a different OGC subpattern in region 3712-3721 having instead the same OGC subpattern as the other clades. Strain PJ-7 is an outlier in the ANI tree compared to the rest of clade V. Region 3712-3721 appears to be ancestral for PJ-7, but the rest of its WTA cassette appears to have been acquired via homologous recombination with a clade V strain or from the same source the ancestral clade V strain acquired its divergent WTA cassette. All clade V strains except PJ-7 have OGC subpattern (3713-3718,9158,3719-3720) in region 3712-3721, whereas strain PJ-7 and all the other clades have OGC subpattern (4723) in region 3712-3721. OGC 3713 has protein orthology to OGC 4723 ( Table 1).

For all clades except clade V, region 3712-3721 has OGC subpattern (4723) and region 3722-3749 begin with OGC subpattern (4724-4730). For the 43 strains in clade I, there are seven OGC patterns in the WTA cassette (Supplementary Table 9, Supplementary Table 8, Table 2). In region 3722-3749, all seven OGC subpatterns begin (4724-4730,5418-5421,4734-4735,5422-5424,4736,4744,5425) but then diverge near the *tarF* gene. There are four OGC subpatterns containing the *tarF* gene: (4738-4741), (5994-5998), (5426-5430), and (7395-7397). For the most part these four OGC subpatterns are unique to clade I and do not appear to be due to recombination. OGC subpatterns (4738-4741) and (5994-5996) are shared by some clade VI strains but in a non-orthologous location and as we discuss below OGCs 4740-4741 appear to be a mis-annotation for clade I strains where a *tarF* gene should be annotated instead. By a non-orthologous location, we mean that the OGCs are not occurring in the same OGC/gene context for the different clades possibly indicating that they are not truly orthologous. After the *tarF* gene, the remainder of region 3722-3749 has the same OGC subpattern (5431-5437) for all clade I strains. As discussed above, there does appear to be recombination in the 3750-3753 region for clade I strains. In the 3754-3756 region, there are two OGC subpatterns: (4754) and (5438). OGC subpattern (5438) occurs in only 5/43 clade I strains, 1/2 clade II strains, and in no other clades. With the limited number of clade II strains it is impossible to guess if OGC subpattern (5438) is ancestral for clade II and recombining with clade I. Alternatively, clade I and clade II may have both acquired OGC subpattern (5438) independently from an unknown source.

For the 35 strains in clade VI, there are seven OGC patterns (Supplementary Table 9, Supplementary Table 8, Table 2). In region 3722-3749, all seven OGC subpatterns begin (4724-4736) but then diverge after the *gtaB* gene but before the *tagH* gene. There are four OGC subpatterns: (10139,4737-4743), (10139,4737,5994-5996,7091-7093,4743), (10139,4737,5994-5996,7091-7093,4743), and () containing no OGCs. For the most part these four OGC subpatterns are unique to clade VI and do not appear to be due to recombination. OGC subpatterns (4738-4741) and (5994-5996) are shared by some clade I strains but in a non-orthologous location as discussed above. The remainder of region 3722-3749 has the same OGC subpattern (4744-4751) for all clade VI strains. As discussed above, there does appear to be recombination in the 3750-3753 region for clade VI strains. In the 3754-3756 region, there is only OGC subpattern (4754).

For clades II, III, IV, and VII recombination based on OGC patterns is harder to evaluate since they only have 2, 3, 1, and 1 strain(s), respectively. For clade VII, the OGCs which are not unique to clade VII are common to most other clades. For clade IV, this is also true with the exception of OGC subpattern (7625-7631) which is shared with clade III but it is unclear if this is due to shared ancestry or recombination. For clade III, OGC patterns are mixed between that seen for clades I and VI with some unique OGCs mixed in but there is no obvious recombination pattern except in region 3754-3756 where there are two OGC subpatterns: (3755) and (4754). For clade II, the OGC patterns are very similar to clade I with some unique OGCs and no evidence of recombination except in region 3754-3756 where there are two OGC subpatterns: (5438) and (4754).

For the eight essential WTA genes that did not have core OGCs, we combined the protein ortholog OGCs ( Table 1) showing: *tuaB*, *mnaA/yvyH*, *tagG*, and *tagH* are in all 108 strains; *tagD*, *tagA*, and *tagB* are in 107 strains (missing from the one clade VII strain); and *tagF* is in only 87 strains. We investigated why *tagF* is missing from 20 strains besides the clade VII strain also missing *tagDAB.* It is also missing from all clade II and III strains and from 15 clade I strains. For the 15 clade I strains two short proteins (OGCs 4740-4741) have homology to *tagF* in nonoverlapping regions but many clade VI strains have both OGCs 4740-4741 and 4746 ( *tagF*). We searched the entire genomes of the strains missing an obvious *tagF* ortholog using tblastn to search at the peptide level the *tag/tarF* medoid proteins for OGCs 4746 ( *tagF*), 5988 ( *tarF*), and 5430 ( *tarF*). For the 15 clade I genomes both 5998 and 5430 matched full length at 65% and 70% identity to a region adjacent to OGC 5431 and overlapping the OGCs 4740 and 4741. This indicates that these 15 genomes are misannotated in this region. Unfortunately, the PGG-based annotation pipeline enforces consistency of annotation but not correctness of annotation. The original RefSeq annotation had OGCs 4740 and 4741 for 24 and 23 strains respectively, while the *tagF* ortholog for the same region was annotated for 15 strains. The PGG refinement process will choose between competing OGC annotation for a region based on predominance which resulted in OGCs 4740 and 4741 replacing the *tagF* ortholog annotation for those 15 strains. This results in 39 genomes being annotated with OGCs 4740 and 4741: 15 from clade I which do not have an alternative *tagF* ortholog and 24 from clade VI which do have an alternative *tagF* ortholog (OGC 4746). In these 24 clade VI strains the entire *tagF* ortholog (1185 bp) from the 15 clade I strains is not present but only the C terminal region (bps 654-1185) which contains OGCs 4740 and 4741 matches with 93% sequence identity. One possibility is that this region was transferred during homologous recombination but due to the presence of another *tagF* gene in the genome this particular *tagF* devolved in these 24 strains leaving two small open reading frames annotated as hypothetical proteins. For the clades II and III genomes no obvious *tagF* ortholog was found using tblastn but significant hits to OGC 5437 ( *tarL*) had low homology and significant but lower homology was seen for several other WTA cassette OGCs. It is not clear whether one or more of these homologs may be providing the needed *tagF* functionality. For the one clade VII strain the entire core WTA machinery is missing ( *tag/tarBADF*). Based on the literature one would expect this to be an engineered strain with limited viability and abnormal cell morphology but it is a soil isolate with normal cell morphology. ^49^ It is an interesting question whether the one clade VII strain is deficient in WTAs but compensating with LTAs.

**Figure 4.**
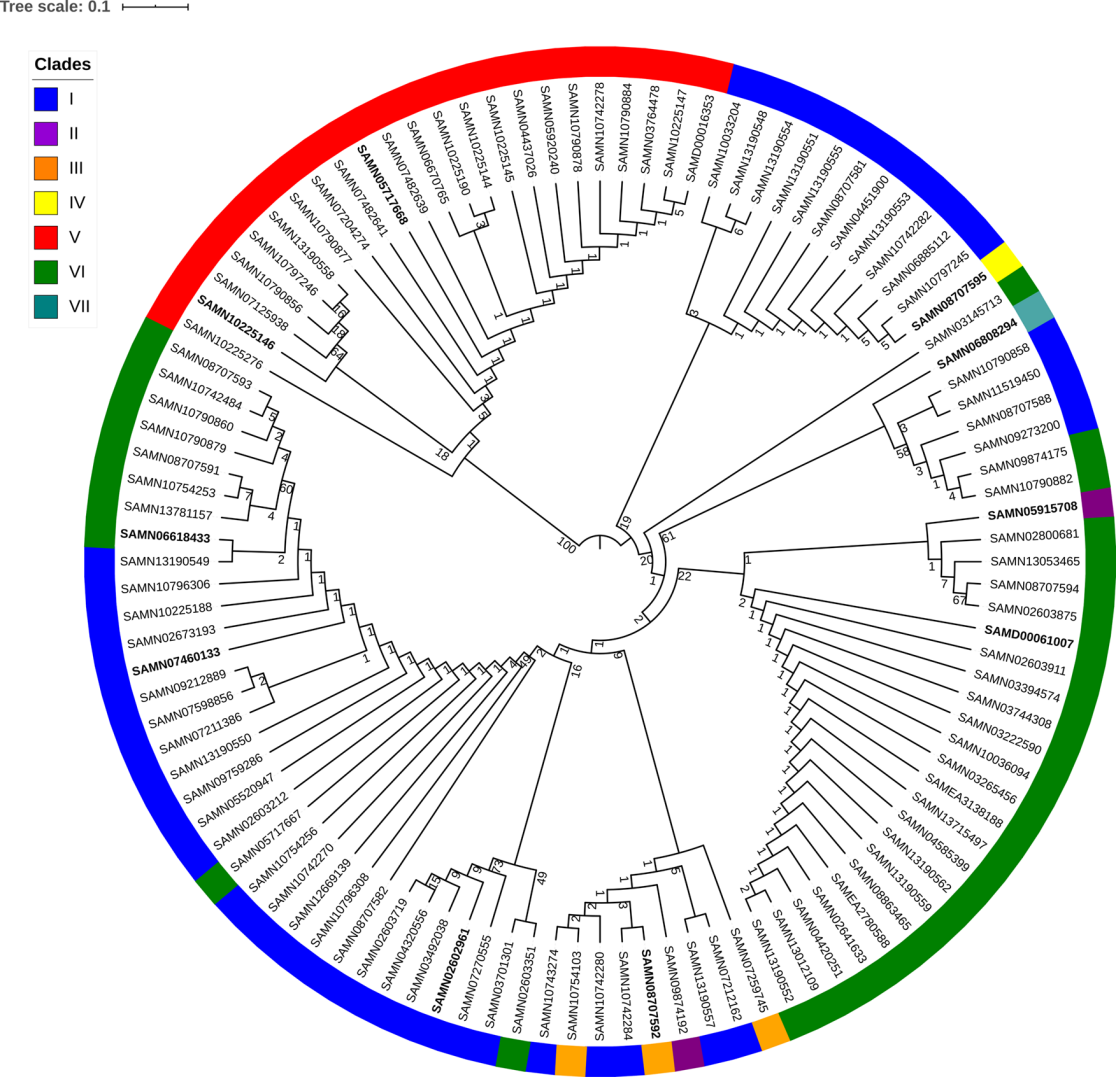
RAxML maximum likelihood tree for trimmed alignment of all 108 WTA cassettes. Midpoint rooted and branchlengths ignored. Strains are color coded by WTA clade membership (I-VII). Numbers at nodes represent bootstrap support. The 10 medoid strains used in Figure 3 are in bold.

To better understand the homologous recombination occurring in the WTA cassettes, we extracted the nucleotide sequences for all 108 cassettes, aligned them, trimmed them to remove columns predominated by gaps, and generated a maximum likelihood tree ( Figure 4). Since clade V strains cluster on one branch of the tree with a much higher bootstrap value than any other branch, they clearly have a very diverged cassette from other strains. The intermixing of other clades in the remainder of the tree possibly indicates active homologous recombination within the WTA cassettes of those clades. The clade V WTA cassette in contrast appears to have been acquired in whole from some more distant organism and is not actively recombining within the WTA cassette with any other strains in other clades. The one exception to this is for strain PJ-7 (SAMN10225146) which does not have region 3712-3721 of the clade V WTA cassette. It is an open question whether this one outlier clade V strain acquired its WTA cassette from some other clade V strain or independently from the same organism as the other clade V strains.

## Discussion

A pangenome analysis of *B. subtilis* ssp *subtilis* indicates that there are seven diverged cassettes of WTA genes (clades I-VII) in *B. subtilis* ssp. *subtilis.* Of these only the type strain glycerol WTA gene cassette, clade VI, is well characterized in the literature, alongside significant work on the ribitol WTA gene cassette of clade I. Based on the ribitol and glycerol specific OGCs three clades (I, II and III) appear to be ribitol WTAs, one clade (VI) appears to be glycerol WTA, and three clades are an indeterminate WTA type. Clade V may be a glycerol WTA based on a longer *tagF* gene which is associated with glycerol WTAs versus a shorter *tagF* for ribitol WTAs. Clade IV may be a ribitol WTA by the same reasoning with a shorter *tagF* gene. Clade VII is missing many key WTA genes ( *tag/tarBADFG*) so it is unclear how the one strain in that clade is constructing WTAs or if it is compensating with LTAs. Additional biochemical characterization of the cell walls for these clades would be required.

More recently it was determined that the ribitol/glycerol distinction of *tar* versus *tag* genes is not distinguishing between *spizizenii* and *subtilis* subspecies but rather either subspecies can contain one or the other. ^34^ Having aligned *tar/tagBAD* genes from multiple *B. subtilis* ssp. *subtilis* strains, Ahn *et al*. ^34^ determined that there were two main clades, one for ribitol WTA (group A) and one for glycerol WTA (group B), with three outliers not in either clade. Group A included four of our clade I strains (BAB-1, UD1022, OH 131.1, BSP1). Group B included four of our clade VI strains (RO-NN-1, BSn5, 168, SG6). One outlier (strain VV2), is one of our clade II strains. Another outlier (strain BEST195), is one of our clade V strains.

The WTA gene cassette is highly variable in *B. subtilis* ssp. *subtilis*, much more so than other essential or core genes. ^6^ This does not appear to be an ancient evolutionary split of strain subtypes, although inheritance is an obvious component, but rather the result of homologous recombination of WTAs as an entire cassette or just regions within the cassette. This method of rapid evolution is often in response to environmental pressure (e.g. phage infection). The WTAs exposed on the cell surface have been shown to be potential phage targets and variation in the WTAs can determine phage susceptibility ^50– 52^ and therefore strain fitness. Likewise, antibiotic susceptibility has been shown to be a driving factor in strain fitness in the wild and WTA biosynthetic proteins are the targets of some antibiotics and further study of these novel WTA OGCs may lead to novel antibiotics. ^53– 57^ WTAs can also modulate specificity of species and strains that can effectively engage in horizontal gene transfer and thereby acquire antibiotic resistance or virulence traits. ^58^ WTAs are also involved in host immune system evasion. ^59– 62^

The high rate of recombination and genetic tractability in *B. subtilis* make it a model organism for biological engineering. Discovery of novel core OGCs using a pan-genome approach can help identify genomic regions capable of excluding gene insertions or non-essential OGCs ready for deletion. The high degree of essential gene conservation in the WTA cassette suggests that they might not be easily deleted. The high level of variation in the WTA cassette is intriguing and a possible target for engineering since it appears to be under adaptive pressure. By naturally rearranging the WTA cassette *B. subtilis* may be able to occupy new niches where acquisition of WTA genes not susceptible to certain antibiotics or phages is advantageous. Conversely, biological engineers might be able to recombine genes conferring antibiotic susceptibility to expand the number of usable antibiotic genes required for manipulating multiple endogenous loci concurrently.

## Conclusion

Reduced sequence conservation of the WTA cassettes in the seven clades we determined may indicate a modified function like the previously documented WTA ribitol/glycerol variation found in two of those clades. This WTA variation poses a number of questions about function, response to environmental pressure, and potential engineering targets as discussed above. An improved understanding of high-frequency recombination of WTA gene cassettes has ramifications for synthetic biology and the use of *B. subtilis* in industry.

## Data availability

### Underlying data

Figshare: Underlying data for ‘Horizontal transfer and evolution of wall teichoic acid gene cassettes in *Bacillus subtilis*’, https://doi.org/10.6084/m9.figshare.14132192.v1

This project contains the following underlying data:

• Table 1. The protein level orthologous OGCs within the WTA cassettes. Column 1 is the gene name/symbol. Column 2 is the set of OGCs determined to be orthologs at the protein level. Column 3 is the number of the 108 strains in the PGG which contain one of the protein level orthologs. Column 4 is OGC medoid sequence RefSeq annotation for one of the protein level orthologs.
• Table 2. OGC subpatterns for the WTA cassettes across clades I-VII. The OGC subpatterns show some limited recombination within the WTA cassettes but most recombination seems limited to the entire cassette. Column 1 is the region between core OGCs within the WTA cassette. Column 2 is an OGC subpattern. Columns 3-9 indicate the number of strains within a clade that has the given OGC subpattern for that row. The rows are ordered relative to their order in the WTA cassette from core OGC 3712 to core OGC 3756.

Data are available under the terms of the Creative Commons Attribution 4.0 International license (CC BY 4.0).

### Extended data

Figshare: Underlying data for ‘Horizontal transfer and evolution of wall teichoic acid gene cassettes in *Bacillus subtilis*’, https://doi.org/10.6084/m9.figshare.14132192.v1

This project contains the following extended data:

• Supplementary Table 1
• Supplementary Table 2
• Supplementary Table 3
• Supplementary Table 4
• Supplementary Table 5
• Supplementary Table 6
• Supplementary Table 7
• Supplementary Table 8
• Supplementary Table 9

Data are available under the terms of the Creative Commons Attribution 4.0 International license (CC BY 4.0).
