# Supplementary material for: Horizontal transfer and evolution of wall teichoic acid gene cassettes in  Bacillus subtilis
Source: F1000Res. 2021 May 7;10:354. [Version 1] doi: 10.12688/f1000research.51874.1 (PMC8753576; doi:10.12688/f1000research.51874.1)
Supplement: Peer review history

# Peer review: Reviewer response for version 1

**DOI:** 10.5256/f1000research.55084.r101224

**Published:** 2022-01-11

**Authors:** Patrick Eichenberger

**Affiliations:** 1 Center for Genomics and Systems Biology, Department of Biology, New York University, New York, NY, USA

This article addresses an interesting question by investigating the evolution of wall teichoic acid (WTA) genes in *B. subtilis*. It is already known that WTA plays a key role in *B. subtilis* during growth; however, the molecular composition of theses anionic polymers vary from strain to strain. For instance, the type strain (168) makes polymers of glycerol, whereas another frequently studied strain (W23) makes polymers of ribitol. By determining the gene composition and patterns of conservation within the cassette encoding the different enzymes in the WTA synthesis pathway, the authors shed light on the evolutionary history of *B. subtilis* strains.

 Unexpectedly, there is no clear phylogenetic distinction between poly-ribitol and polyglycerol producing strains, thus strongly suggesting that horizontal gene transfer and recombination within the WTA locus is frequent. As already mentioned by the original reviewer, ample support is provided in the article for this hypothesis. As suggested in the previous round of review, the authors have now added an Average Nucleotide Identity (ANI) analysis, whose outcome is also consistent with the expectations.

 The methods are explained in detail and with clarity. My only suggestion would be to rephrase the following statement on p.3 “Curiously, eight of the 305 essential genes that were not core genes”. If I understood correctly, of the 305 essential genes, only 8 are not core genes, but the sentence seems to imply that none of the 305 essential genes were core genes and that the focus of the paper will be on 8 of these genes.

Is the work clearly and accurately presented and does it cite the current literature?

Yes

If applicable, is the statistical analysis and its interpretation appropriate?

Yes

Are all the source data underlying the results available to ensure full reproducibility?

Yes

Is the study design appropriate and is the work technically sound?

Yes

Are the conclusions drawn adequately supported by the results?

Yes

Are sufficient details of methods and analysis provided to allow replication by others?

Yes

Reviewer Expertise:

Genomics and molecular biology of endospore-forming bacteria

I confirm that I have read this submission and believe that I have an appropriate level of expertise to confirm that it is of an acceptable scientific standard.

# Peer review: Reviewer response for version 1

**DOI:** 10.5256/f1000research.55084.r101882

**Published:** 2021-12-16

**Authors:** José Escudero

**Affiliations:** 1 Departamento de Sanidad Animal and VISAVET, Complutense University of Madrid (Universidad Complutense de Madrid), Madrid, Spain

In this manuscript the authors report discrepancies between the core genome of B. subtilis and its set of essential genes. Some of the genes that do not match encode the WTA synthesis pathways, which are essential. The reason for the discrepancies, they argue, is that horizontal gene transfer and homologous recombination have led to extensive exchange in these genes leading some alleles not to be recognised as the same OGC and hence not be considered part of the core genome. This type of mosaicism has been described in *S. pneumoniae* quinolone resistance determining regions (QRDRs) by the group of De la Campa, so I believe maybe mentioning it would be relevant to provide context.

 I find this work to be well carried out and show results that are relevant. I nevertheless find it challenging to follow and believe it would benefit from extensive rewriting to make the language less specialised. Rewriting would especially benefit the results section where a higher context story, rather than a point by point enumeration of results, could be easier to understand, and the details would still be available in the table provided. I would also suggest to include an introductory figure showing, among other things, the basic genetic structure of the WTA genes in a type strain such as Bs168. Maybe including *subtilis *and *spizizenii *subspecies to compare these sets of genes would be even better. 

 I would also suggest to change the figure legend titles to sentences telling the result rather than describing the figure and methodology. The description and methodology can go in the figure legend after the title.

 Altogether, I believe that the results are relevant but need to be told in a more clear way to reach a broader audience.

Is the work clearly and accurately presented and does it cite the current literature?

Partly

If applicable, is the statistical analysis and its interpretation appropriate?

I cannot comment. A qualified statistician is required.

Are all the source data underlying the results available to ensure full reproducibility?

Yes

Is the study design appropriate and is the work technically sound?

Yes

Are the conclusions drawn adequately supported by the results?

Yes

Are sufficient details of methods and analysis provided to allow replication by others?

Yes

Reviewer Expertise:

Antibiotic resistance, horizontal gene transfer, integrons, protein evolution, recombination

I confirm that I have read this submission and believe that I have an appropriate level of expertise to confirm that it is of an acceptable scientific standard, however I have significant reservations, as outlined above.

# Peer review: Reviewer response for version 1

**DOI:** 10.5256/f1000research.55084.r86085

**Published:** 2021-06-18

**Authors:** Jeffrey B. Jones

**Affiliations:** 1 Department of Plant Pathology, University of Florida, Gainesville, FL, USA

The manuscript is well written and provides ample support for evolution within the WTA gene cassette. Although the authors have provided considerable information on the relationships of the genes in the different clades, I wonder if it would be appropriate to provide an ANI matrix comparing all strains to show relationships between the clades and the ANI values between strains. I suspect although I may be incorrect that these clades may strongly relate to the relative ANI values. It may help in explaining the extent of recombination or perhaps refute that idea. I am more familiar with ANI values for Gram negative bacteria and although values around 97 are relatively close values above 99% show much tighter relationships and may help in explaining the extent of recombination. Although they state the importance of these findings for synthetic biology using this bacterium, it would be helpful if they would provide more information on how.

Is the work clearly and accurately presented and does it cite the current literature?

Yes

If applicable, is the statistical analysis and its interpretation appropriate?

I cannot comment. A qualified statistician is required.

Are all the source data underlying the results available to ensure full reproducibility?

Yes

Is the study design appropriate and is the work technically sound?

Yes

Are the conclusions drawn adequately supported by the results?

Yes

Are sufficient details of methods and analysis provided to allow replication by others?

Yes

Reviewer Expertise:

plant-microbe interactions, bacterial pathogenesis

I confirm that I have read this submission and believe that I have an appropriate level of expertise to confirm that it is of an acceptable scientific standard, however I have significant reservations, as outlined above.

# Response

**Published:** 2021-06-21

**Authors:** Granger Sutton

**Affiliations:** J. Craig Venter Institute, USA

Thank you for the feedback. We're not sure exactly what form of ANI matrix you are requesting. We provide a whole genome all versus all ANI matrix for the 108 genomes as Supplementary Table 6 and Figure 2 shows the tree based on this matrix. Perhaps you want us to highlight this more in the text? We will attempt to be more explicit about applications for synthetic biology in our revision.
